# Non-canonical function of FIP200 is required for neural stem cell maintenance and differentiation by limiting TBK1 activation and p62 aggregate formation

**DOI:** 10.1038/s41598-021-03404-7

**Published:** 2021-12-13

**Authors:** Hang Liu, Chenran Wang, Fei Yi, Syn Yeo, Michael Haas, Xin Tang, Jun-Lin Guan

**Affiliations:** Department of Cancer Biology, University of Cincinnati College of Medicine, Cincinnati, OH 45267 USA

**Keywords:** Cell biology, Stem cells

## Abstract

FIP200 is an essential autophagy gene implicated in the regulation of postnatal neural progenitor/stem cells (NSCs). However, the contribution of FIP200’s canonical-autophagy function and its non-canonical functions to postnatal NSC maintenance remains unclear. Utilizing a recently generated *Fip200-4A* allele that specifically impairs FIP200’s canonical-autophagy function, we found that non-canonical functions of FIP200 was required for regulation of mouse NSC maintenance and neurogenesis in vivo. Ablating the non-canonical functions of FIP200, but not its autophagy function, increased TBK1 activation and p62 phosphorylation at S403 in NSCs. Phosphorylation of p62 was dependent on TBK1 kinase activity and increased the propensity of p62 aggregate formation specifically in FIP200-null NSCs. Accordingly, inhibition of TBK1 by amlexanox reduced p62 aggregates and restored NSC maintenance and differentiation in *Fip200*^*hGFAP*^ cKO mice. These results reveal a mechanism for the non-canonical functions of FIP200 in NSC maintenance and differentiation by limiting TBK1 activation and subsequently, p62 aggregate formation.

## Introduction

Like stem cells in many tissues, neural progenitor/stem cells (NSCs) are responsible for the generation of new neurons and glial cells in the brain for tissue homeostasis and after injuries^1–4^. In adult brains, NSCs reside primarily within the subventricular zone (SVZ) of the lateral ventricles^5^ and the subgranular zone (SGZ) of the hippocampal dentate gyrus (DG)^6^, where relatively quiescent adult NSCs give rise to transit-amplifying progenitor cells which contribute to lineage-restricted neuroblasts, generating new neurons for the maintenance and reorganization of the existing circuitry^7,8^. Deficiency in NSC maintenance and/or neurogenesis contributes to both developmental defects and neurodegenerative disorders during aging such as Alzheimer’s, Parkinson’s, and Huntington’s diseases. Thus, understanding the molecular and cellular mechanisms of NSC maintenance and neurogenesis is important for developing potential treatments for these diseases.

Autophagy is an evolutionarily conserved cellular process that mediates the clearance of cytosolic proteins and organelles. Autophagy dysfunction is implicated in a variety of diseases such as cancers, autoimmune diseases and neurodegenerative diseases^9–12^. It plays an especially critical role in post-mitotic cells such as neurons, which cannot dilute protein aggregates and damaged organelles through cell division. Indeed, multiple studies have shown that deletion of different autophagy genes such as Atg5, Atg7 and FIP200 in neurons caused increased ubiquitinated protein aggregates and neurodegenerative defects^13–15^. In contrast to observations in neurons, however, we found recently that whereas deletion of FIP200 led to the gradual depletion of NSCs and their defective neurogenesis, NSCs tolerated the loss of other autophagy genes including Atg5, Atg7 and Atg16L1 without exhibiting any apparent phenotypes^16,17^. Previous studies showed that besides its essential role in the ULK1/Atg13/FIP200/Atg101 complex for autophagy induction, FIP200 interacts with several other proteins to regulate diverse cellular functions^18^. However, the potential roles and mechanisms in the regulation of NSCs by such non-canonical functions of FIP200 are not well characterized.

p62/SQSTM1 is a major cargo receptor for the selective degradation of ubiquitinated protein aggregates by autophagy^19–23^. Mutations in p62 have also been associated with neurodegenerative diseases such as amyotrophic lateral sclerosis (ALS) and frontotemporal lobar degeneration (FTLD)^24^. Recent studies revealed that these mutations disrupted selective autophagy and reduced anti-oxidative stress response to contribute to the neurotoxicity in the diseases^25^. Conversely, blocking autophagy leads to increased accumulation of p62 in the same ubiquitinated protein aggregates in neurons and other cells^10,15,19,26^. Nevertheless, the significance of the increased p62 aggregates in neurons are not clear, as p62 ablation did not rescue neuronal degenerative phenotype induced by Atg7 cKO^26^. Interestingly, unlike other regions of the brain, p62 aggregates were detected in NSCs after deletion of FIP200, but not Atg5, Atg16L1, or Atg7, and deletion of p62 rescued NSC defects in Fip200 cKO mice, suggesting a critical role of p62 aggregate formation in mediating defective NSC maintenance and neurogenesis^17^.

To address the potential role of non-canonical functions of FIP200 in the regulation of NSC and other biological processes by rigorous genetic analysis in vivo, we recently identified residues 582–585 (LQFL) in FIP200 for its interaction with Atg13, and generated a mutant knock-in mice with these residues mutated to AAAA (i.e. FIP200-4A mutant)^27^ that specifically disrupted FIP200 canonical function in autophagy induction. Analysis of FIP200-4A mutant in fibroblasts and mammary tumor cells showed that FIP200 deletion, but not loss of its autophagy function, led to the activation of TBK1 (TANK-binding kinase 1)^28^. Similar to p62, mutations in TBK1 was identified as causal for ALS and FTLD^29,30^. Other studies showed that TBK1 can phosphorylate p62 at S403 to promote p62 binding to ubiquitin and p62-ubiquitin aggregates formation^31–35^. Further, both p62 and TBK1 mutations linked to ALS-FTLD also compromise selective autophagy for the removal of ubiquitinated protein aggregates and cause neurotoxicity^25^. Thus, emerging evidence suggests a potential role for TBK1 to mediate the regulation of p62/ubiquitinated protein aggregates by non-canonical functions of FIP200 in NSCs.

In this study, we used genetic approaches to abrogate FIP200’s autophagy function specifically using the recently generated *Fip200-4A* allele to investigate the mechanisms of the differential effects of deletion of *Fip200* versus other autophagy genes in NSCs in vivo. We found that non-canonical functions of FIP200 was required for the regulation of NSC maintenance and neurogenesis in vivo. Loss of non-canonical functions of FIP200 but not its autophagy function increased TBK1 activation and p62 phosphorylation in NSCs. TBK1 phosphorylation of p62 increased its aggregates formation specifically in FIP200-null NSCs, whereas inhibiting TBK1 reduced p62 aggregates and restored NSC maintenance and differentiation in *Fip200*^*hGFAP*^ cKO mice. These results reveal a mechanism for the non-canonical functions of FIP200 to regulate NSC maintenance and differentiation by limiting TBK1 activation and its phosphorylation of p62 for ubiquitin aggregates formation.

## Results

### Non-canonical autophagy function of FIP200 is required for the maintenance and differentiation of NSCs

**Figure 1:**
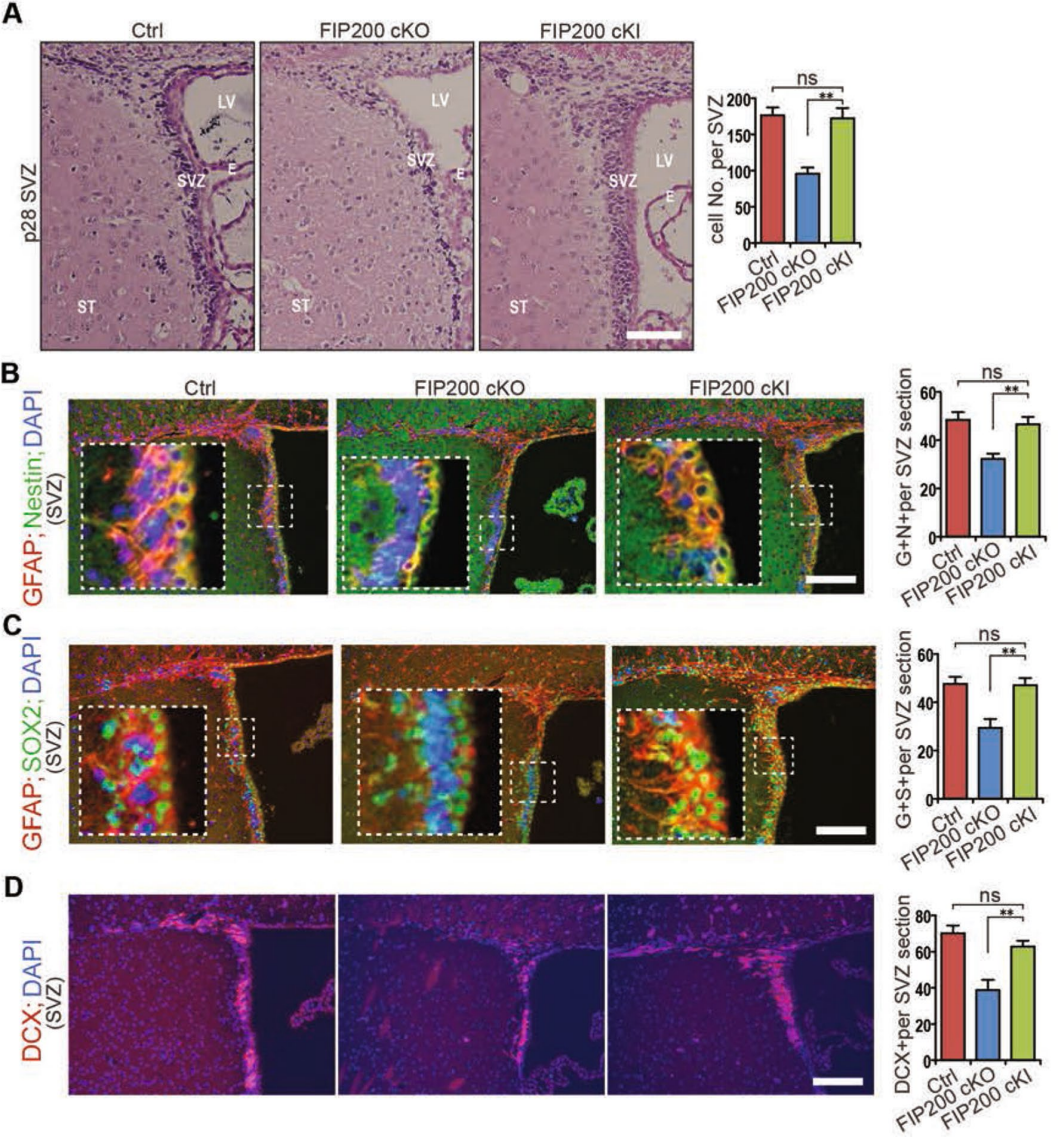
Non-canonical function of FIP200 is required for NSC maintenance and neurogenesis. (**A**) H&E staining of the SVZ from control (Ctrl), *Fip200*^*hGFAP*^ cKO and *Fip200*^*hGFAP*^ cKI mice at P28. Mean ± SEM of SVZ cell numbers is shown on the right (n = 3 mice each). (**B**–**D**) Immunofluorescence for GFAP and Nestin (**B**), GFAP and SOX2 (**C**), and DCX (**D**) in the SVZ from Ctrl, *Fip200*^*hGFAP*^ cKO and *Fip200*^*hGFAP*^ cKI mice at P28. Boxed areas showed more details. Mean ± SEM of positively stained cells are shown on the right (n = 3 mice each). Bars: 100 µm. **, *P* < 0.01.

In previous studies, we created NSC-specific ablation of several autophagy genes including *Fip200*, *Atg5* and *Atg16L1* to assess the role of autophagy and potential non-canonical functions of these genes in the regulation of NSC maintenance and neurogenesis. We found that deletion of FIP200 (involved in autophagy induction), but not Atg5, Atg7 or Atg16L1 (required for autophagosome elongation) compromised NSC maintenance and neurogenesis^17^. To further explore the unique role of FIP200 in NSCs, we created and analyzed NSC-specific FIP200-4A mutant conditional knock-in mice (designated as *Fip200*^*hGFAP*^ cKI mice) to directly assess the effect of disrupting FIP200-mediated autophagy on NSCs in vivo. We first performed H/E staining of SVZ sections from P28 *Fip200*^*hGFAP*^ cKI as well as Ctrl and *Fip200*^*hGFAP*^ cKO mice. As observed previously, we found a significant decrease of the number of SVZ cells in *Fip200*^*hGFAP*^ cKO mice (Fig. 1A). In contrast, *Fip200*^*hGFAP*^ cKI mice showed comparable numbers of SVZ cells as Ctrl mice, suggesting that specific disruption of autophagy function of FIP200 did not result in depletion of NSCs. Double immunofluorescent staining for NSC markers GFAP/Nestin (Fig. 1B), which could distinguish mature astrocytes from stem cells, and progenitor cells marker GFAP/SOX2 (Fig. 1C) provided further support for normal NSC maintenance in *Fip200*^*hGFAP*^ cKI mice. We also examined NSC differentiation and found similar levels of neurogenesis in *Fip200*^*hGFAP*^ cKI mice as determined by DCX staining (Fig. 1D). These results suggest that, rather than potentially differential functions of autophagy induction (FIP200) and autophagosome elongation (Atg5 and Atg16L1), it is likely that the loss of non-canonical functions of FIP200 upon its deletion is responsible for the defective NSC maintenance and neurogenesis in *Fip200*^*hGFAP*^ cKO mice.

### Increased TBK1 activation and p62 phosphorylation upon loss of non-canonical functions of FIP200 in NSCs

**Figure 2:**
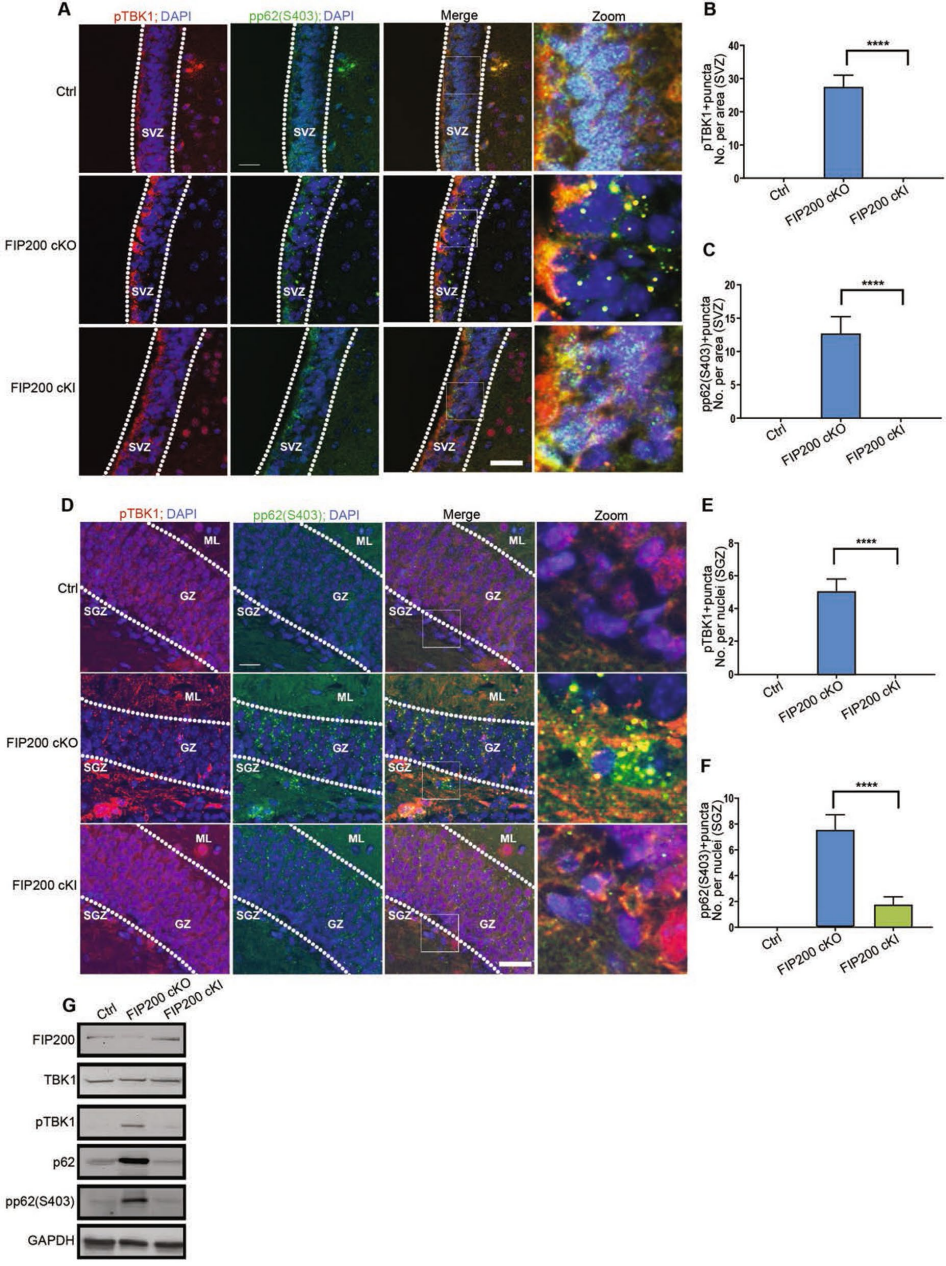
Loss of Non-canonical function of FIP200 induces phosphorylation of TBK1 and phospho-p62 (S403) in NSCs. (**A**–**F**) Immunofluorescence for phosphorylated TBK1 and phosphorylated p62 (S403) in the SVZ (**A**–**C**) and SGZ (**D–F**) of Ctrl, *Fip200*^*hGFAP*^ cKO and *Fip200*^*hGFAP*^ cKI mice at P28. Dotted lines indicate the boundaries of the SVZ (**A**) and GZ (**D**). Mean ± SEM of pTBK1 (**B**,**E**) and pp62 (**C**,**F**) puncta numbers per SVZ area (**B**,**C**) and SGZ section (**E**,**F**) (n = 5 mice each). Bars: 50 µm. ****, *P* < 0.0001. (**G**) Lysates extracted from neurospheres of Ctrl, *Fip200*^*hGFAP*^ cKO and and *Fip200*^*hGFAP*^ cKI mice, and analyzed by western blot using various antibodies as indicated.

Besides its role in the ULK1/Atg13/FIP200/Atg101 complex for autophagy induction, FIP200 has been shown to interact with several other signaling molecules such as FAK, Tsc1, p53 and TBK1^18,27,36^. Interestingly, recent studies showed that FIP200 deletion led to TBK1 activation in mammary tumor and other cells^28,37^. However, we found that loss of FIP200 autophagy function by FIP200-4A mutation did not induce TBK1 activation in mammary tumor cells, suggesting that TBK1 may mediate non-autophagy functions of FIP200 in these cells^28^. To determine the relative contribution of the loss of autophagy and non-canonical functions of FIP200 in TBK1 activation in NSCs, we examined TBK1 activation in the SVZ and DG of *Fip200*^*hGFAP*^ cKO and *Fip200*^*hGFAP*^ cKI mice. Increased TBK1 phosphorylation in puncta were found in the SVZ and DG of *Fip200*^*hGFAP*^ cKO mice, but not *Fip200*^*hGFAP*^ cKI mice (Fig. 2A,B,D,E). Recent studies showed that TBK1 can phosphorylate p62 at S403 to promote p62 binding to ubiquitin for the removal of p62-ubiquitin aggregates via selective autophagy^33,34^. Thus, TBK1 activation upon the loss of non-canonical functions of FIP200 could trigger more p62 aggregate formation in FIP200-null NSCs due to blockade of selective autophagy, which has been linked to the defective NSC phenotypes in *Fip200*^*hGFAP*^ cKO mice^17^. Indeed, we found increased aggregates of phosphorylated p62 in the SVZ and DG of *Fip200*^*hGFAP*^ cKO mice, but not *Fip200*^*hGFAP*^ cKI mice, which were co-localized with the phosphorylated TBK1 puncta (Fig. 2A,C,D,F). To further validate the specific increase in TBK1 activation and p62 phosphorylation upon the loss of non-autophagy functions of FIP200, we quantified their levels in neurospheres prepared from SVZ cells of *Fip200*^*hGFAP*^ cKO, *Fip200*^*hGFAP*^ cKI and Ctrl mice. Consistent with observations in vivo, the levels of TBK1 activation, total p62 and phosphorylated p62 were increased in neurospheres from *Fip200*^*hGFAP*^ cKO, but not *Fip200*^*hGFAP*^ cKI mice, relative to that from Ctrl mice (Fig. 2G). Together, these results suggest that TBK1 activation and its phosphorylation of p62 are induced upon the specific loss of non-autophagy functions of FIP200, which could lead to p62 aggregates formation and defective NSC maintenance and differentiation.

### TBK1 activation and its phosphorylation of p62 increase p62 aggregates formation upon FIP200 deletion

**Figure 3:**
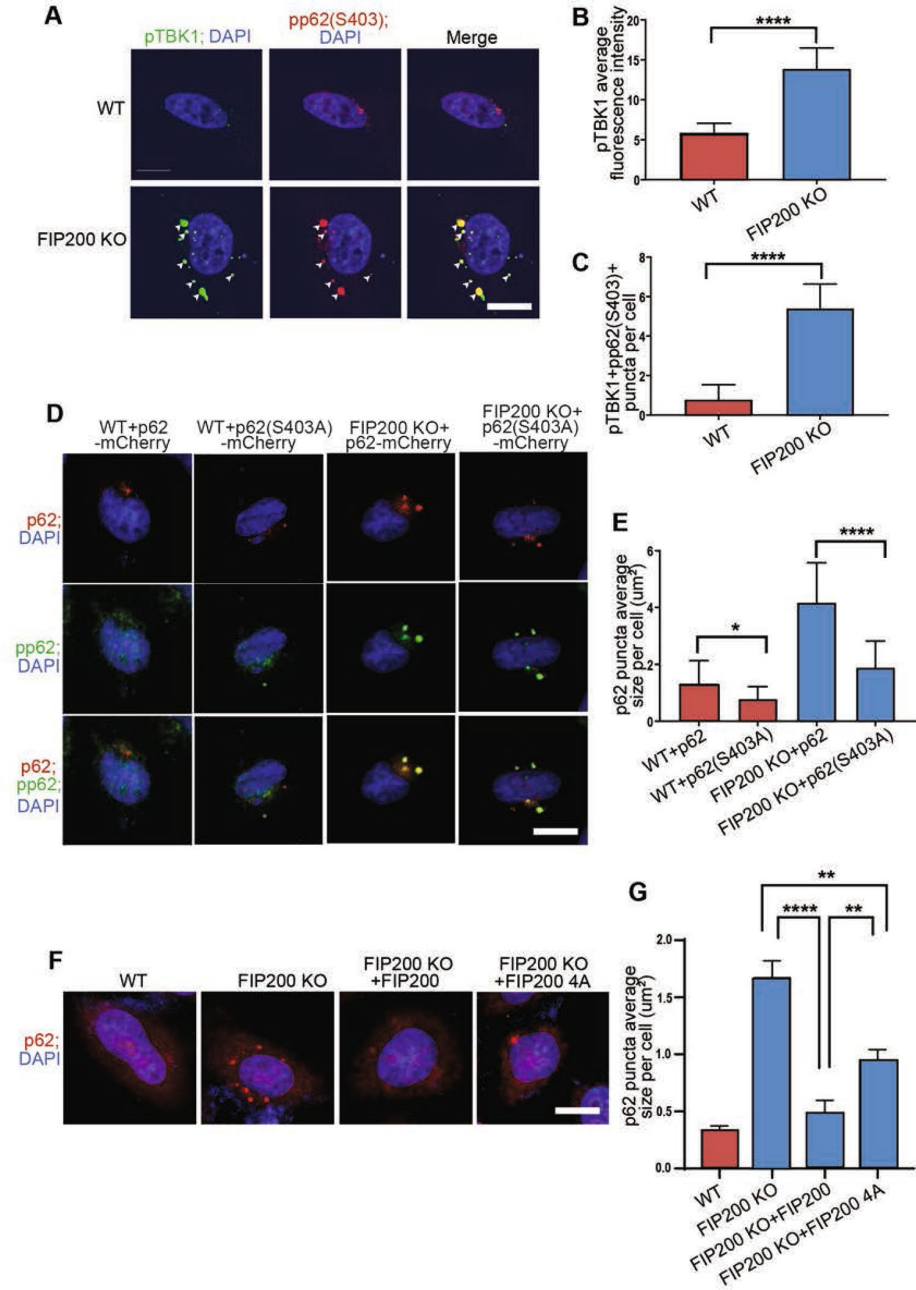
TBK1 phosphorylation of p62 at S403 promotes its aggregates formation in FIP200 KO cells specifically. (**A**) Representative immunofluorescence images for phosphorylated TBK1 and p62 (S403) in WT and FIP200 KO HeLa cells. (**B**,**C**) Quantification of (**B**) pTBK1 + fluorescence intensity per cell and (**C**) pp62(403) + and pTBK1 + puncta number per WT and FIP200 KO cell. (**D**) Representative immunofluorescence images for mCherry fused p62 and p62 (S403A) mutant as well as their phosphorylated versions (pp62 and pp62(S403A) mutant) in WT and FIP200 KO HeLa cells, respectively. (**E**) Quantification of p62 and p62 (S403A) puncta size per WT and FIP200 KO cell. (**F**) Representative immunofluorescence images of p62 in WT and FIP200 KO HeLa cells expressing empty Vector, FIP200 or FIP200-4A. (**G**) Quantification of p62 + puncta size per cell in (**F**). Scale bars: 10 µm. *, *P* < 0.05; **, *P* < 0.01; ****, *P* < 0.0001. Graphs represent mean ± SEM.

**Figure 4:**
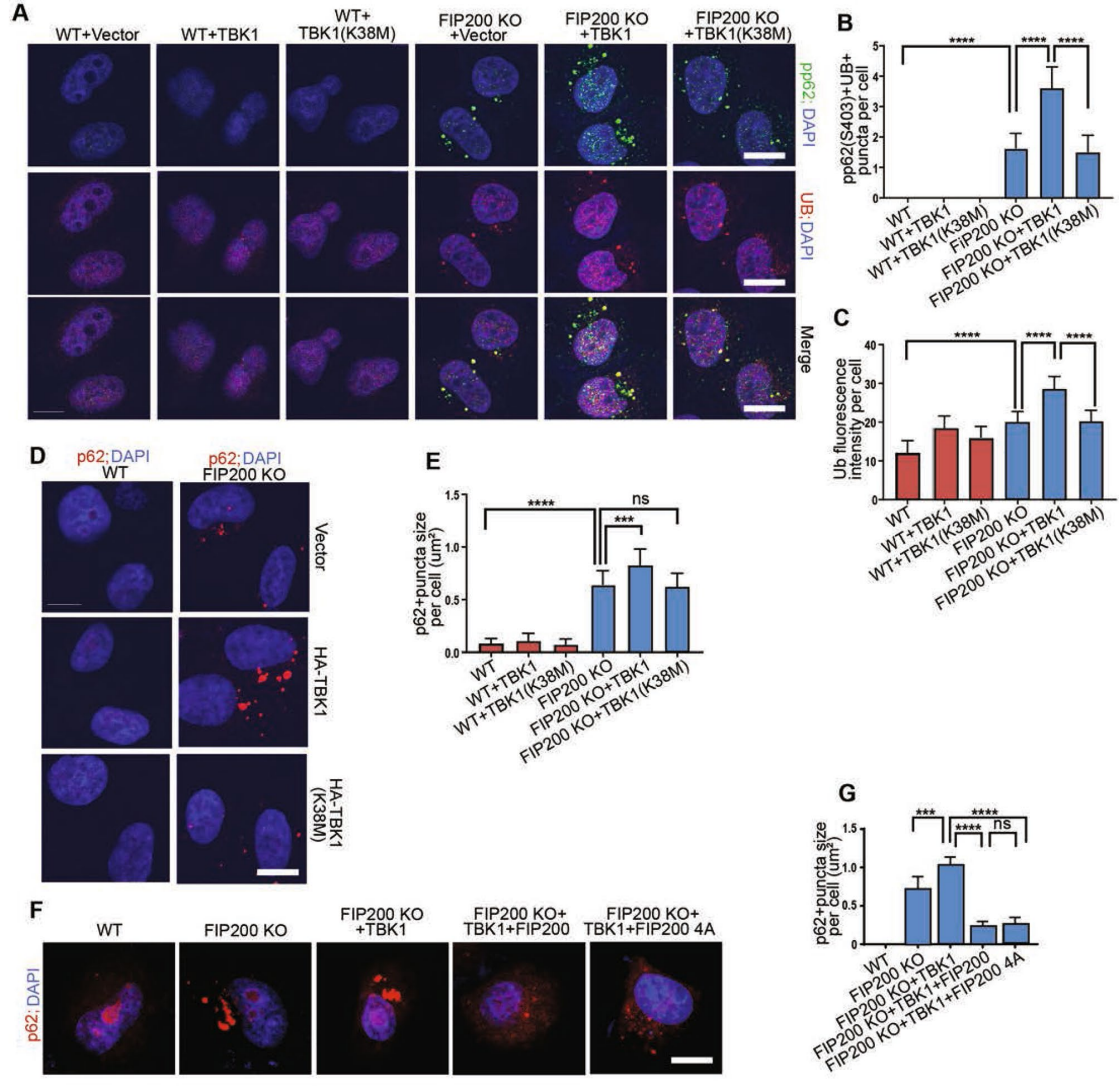
TBK1 kinease dead mutation suppresses the accumulation of p62 puncta. (**A**) Immunofluorescence images of phosphorylated p62 (S403) and ubiquitin in WT and FIP200 KO HeLa cells expressing empty Vector, TBK1 or kinase dead TBK1(K38M). (**B**,**C**) Quantification of fluorescence intensity for (**B**) phosphorylated p62 (S403) and (**C**) ubiquitination per WT and FIP00 KO HeLa cells in (**A**). (**D**) Immunofluorescence images of p62 in WT and FIP200 KO HeLa cells expressing empty vector, TBK1 and kinase dead TBK1(K38M). (**E**) Quantification of puncta size for p62 per WT and FIP00 KO HeLa cells in (**D**). (**F**) Immunofluorescence images of p62 in WT and FIP200 KO HeLa cells expressing various vectors as indicated. (**G**) Quantification of p62 + puncta size per cell in (**F**). Scale bars: 10 µm. ***, *P* < 0.001; ****, *P* < 0.0001. ns, no significance. Graphs represent mean ± SEM.

**Figure 5:**
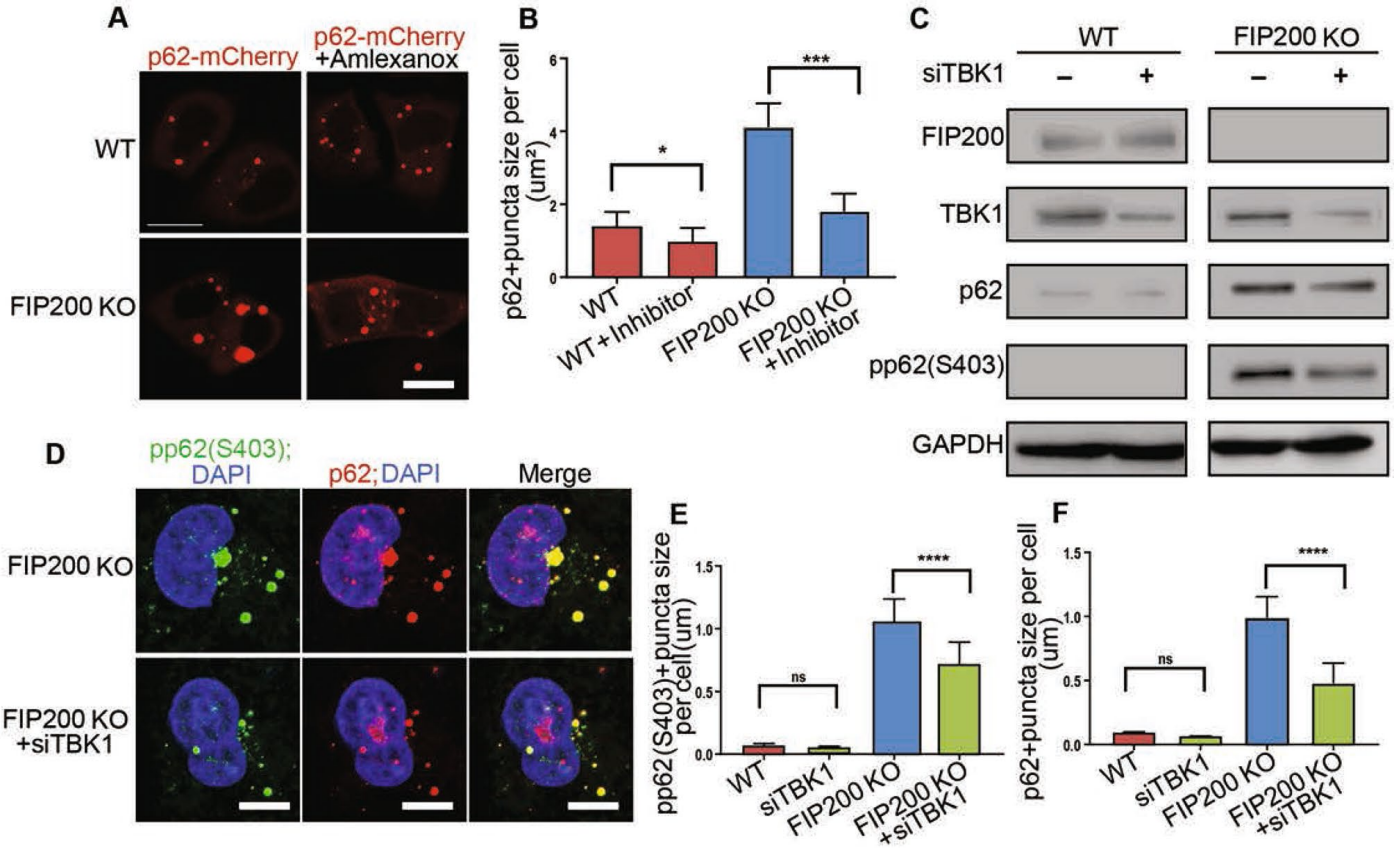
Inhibition of TBK1 suppresses the accumulation of p62 puncta. (**A**) Immunofluorescence images of mCherry fused p62 in WT and FIP200 KO HeLa cells treated with or without 200 µM Amlexanox. (**B**) Quantification of size of mCherry-p62 labeled puncta in WT and FIP200 KO HeLa cells treated with or without Amlexanox. (**C**) Cropped immunoblots showing levels of FIP200, TBK1, p62, pp62 (S403) and GAPDH in WT and FIP200 KO HeLa cells with or without TBK1 siRNA transfection. (**D**) Immunofluorescence images of pp62 (S403) and p62 in FIP200 KO HeLa cells with or without TBK1 siRNA transfection. (**E**,**F**) Quantification of phosphorylated (**E**) p62 (S403) + and (**F**) p62 + puncta size per WT or FIP200 KO cell with or without TBK1 siRNA transfection. Scale bars: 10 µm. *, *P* < 0.05; ***, *P* < 0.001. Graphs represent mean ± SEM.

To evaluate the potential role of TBK1 in promoting p62 aggregates formation after FIP200 deletion, we next examined phosphorylation status of TBK1 and p62 in FIP200 KO HeLa cells. Similar to observations in NSCs, increased numbers of puncta containing co-localized activated TBK1 and phosphorylated p62 were found in FIP200 KO HeLa cells versus WT HeLa cells (Fig. 3A–C). Further, ectopic expression of p62 with mCherry tag (p62-mCherry) also led to the formation of larger p62 as well as phosphorylated p62 puncta in FIP200 KO HeLa cells than that expressed in WT HeLa cells (Fig. 3D,E). p62 mutant converting S403 to A reduced its ability to induce larger puncta in FIP200 KO HeLa cells, supporting a role for TBK1 phosphorylation at S403 to promote p62 aggregates formation, consistent with previous observations^33,34^ (Fig. 3D,E). As expected, re-expression of FIP200 eliminated p62 puncta in FIP200 KO cells (Fig. 3F,G). Re-expression of FIP200-4A mutant also decreased p62 puncta, although not to the same extent as re-expression of FIP200, in these cells. We also found that ectopic expression of TBK1, but not a kinase dead mutant (K38M), further increased p62 phosphorylation and ubiquitination in FIP200 KO HeLa cells (Fig. 4A–C). More importantly, larger and more p62 puncta were observed with overexpression of TBK1, but not K38M mutant, specifically in FIP200 KO HeLa cells (Fig. 4D,E). Moreover, re-expression of FIP200 and FIP200-4A mutant both significantly reduced p62 puncta formation induced by ectopic expression of TBK1 in FIP200 KO cells to similar levels (Fig. 4F,G), supporting the hypothesis that the TBK1 induced increase in p62 puncta formation was correlated with the loss of non-canonical functions of FIP200. Consistent with this, treatment of FIP200 KO HeLa cells with TBK1 inhibitor Amlexanox reversed the increased p62 puncta size in these cells (Fig. 5A,B). Similarly, TBK1 knockdown by siRNA also reduced puncta size of p62 and phosphorylated p62 in FIP200 KO HeLa cells (Fig. 5C–F). Together, these results provide further support that activated TBK1 can phosphorylate p62 to promote its aggregates formation after FIP200 deletion, which was dependent on FIP200 non-canonical autophagy function.

### TBK1 inhibition alleviates p62 aggregates and restores NSC maintenance and differentiation in FIP200 cKO mice

**Figure 6:**
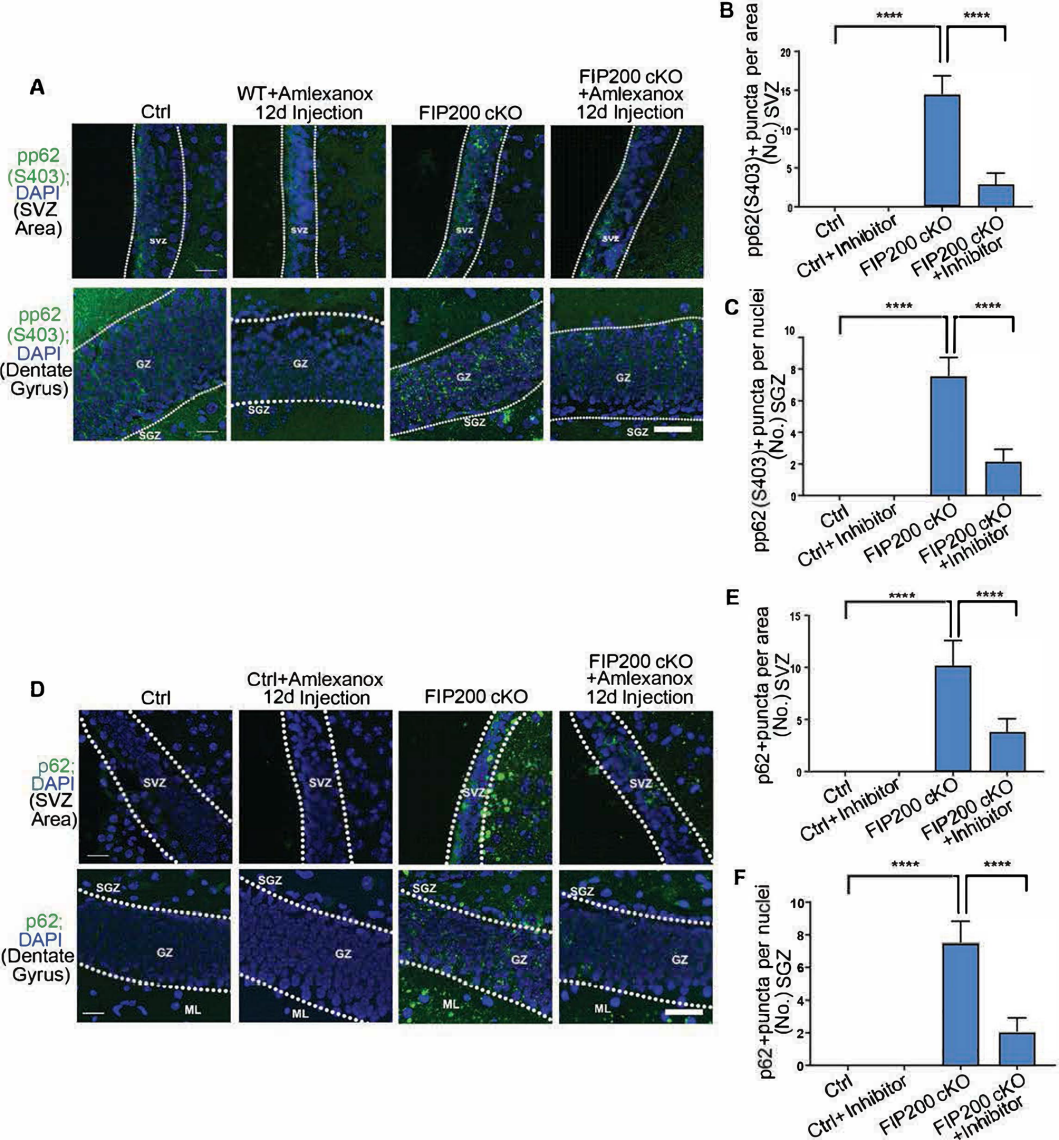
TBK1 inhibitor treatment suppresses the accumulation of p62 puncta in the SVZ and SGZ of *Fip200*^*hGFAP*^ cKO mice. (**A**) Immunofluorescence images of phosphorylated p62(S403) in the SVZ and DG of Ctrl and *Fip200*^*hGFAP*^ cKO mice with or without Amlexanox treatment. (**B**,**C**) Quantification of P62 + puncta per area in (**B**) SVZ and (**C**) per nuclei in SGZ of Ctrl and *Fip200*^*hGFAP*^ cKO mice with or without Amlexanox treatment. Dotted lines indicate the boundaries of the SVZ and GZ. (n = 3 mice each). (**D**) Immunofluorescence images of p62 in SVZ and DG of Ctrl and *Fip200*^*hGFAP*^ cKO mice with or without Amlexanox treatment. (**E**,**F**) Quantification of P62 + puncta per area in (**E**) SVZ and (**F**) per nuclei in SGZ of Ctrl and *Fip200*^*hGFAP*^ cKO mice with or without Amlexanox treatment. Dotted lines indicate the boundaries of the SVZ and GZ. (n = 3 mice each). Scale bars: 50 µm. ***, *P* < 0.001. Graphs represent mean ± SEM.

**Figure 7:**
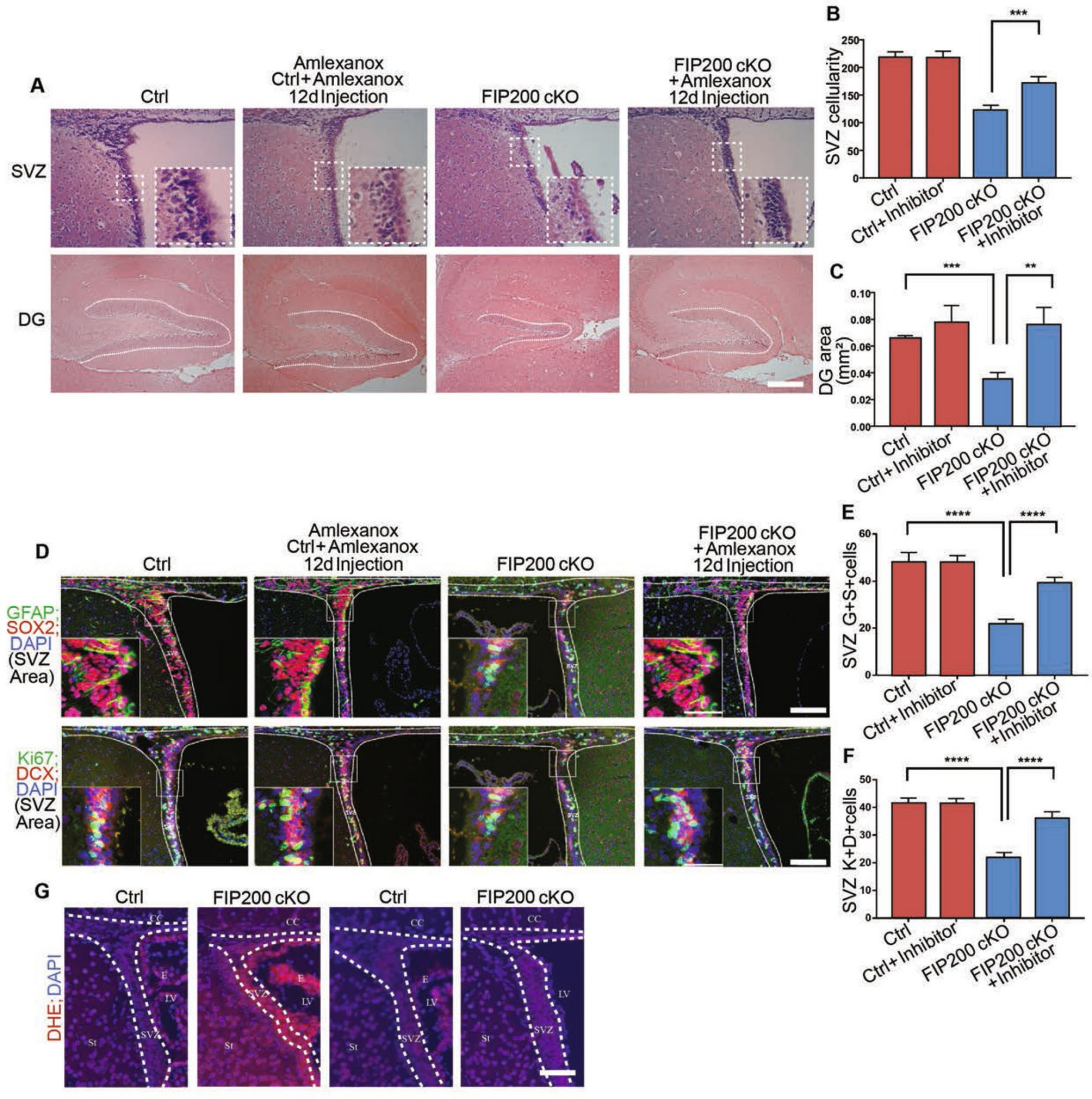
TBK1 inhibitor treatment reduces the increased superoxide in *Fip200*^*hGFAP*^ cKO mice. (**A**) H&E staining of SVZ and DG from Ctrl and *Fip200*^*hGFAP*^ cKO mice with or without the treatment of Amlexanox at P28. Dotted lines indicate the SVZ (Scale bars: 100 µm) and DG (Scale bars: 200 µm) boundaries. Boxed areas indicated more details. (**B**,**C**) Quantification of (**B**) SVZ cellularity and (**C**) DG area per section (n = 5 mice each). Dotted lines indicate the SVZ and DG boundaries. (**D**) Immunofluorescence images for GFAP + ;SOX2 + and Ki67 + ;DCX + in SVZ from Ctrl and *Fip200*^*hGFAP*^ cKO mice at P28. Dotted lines indicate the SVZ boundaries. Scale bars: 100 µm. Boxed areas indicated more details. (**E**,**F**) Quantification of (**E**) GFAP + and SOX2 + and (**F**) Ki67 + and DCX + cell number per SVZ section. (n = 3 mice each). (**G**) Immunofluorescence images of DHE in SVZ of Ctrl and *Fip200*^*hGFAP*^ cKO mice with or without Amlexanox treatment. Scale bars: 100 µm. ***, *P* < 0.001. Graphs represent mean ± SEM.

To evaluate directly a role for TBK1 activation in inducing p62 aggregates and NSC defects, we examined pharmacological inhibition of TBK1 in *Fip200*^*hGFAP*^ cKO mice. We treated Ctrl and *Fip200*^*hGFAP*^ cKO mice with the TBK1 inhibitor, amlexanox, by daily intraperitoneal injection for 12 days (25 mg per kg (body weight)). TBK1 inhibition decreased p62 phosphorylation (Fig. 6A–C) as well as p62 aggregates formation (Fig. 6D–F) in the SVZ and DG of *Fip200*^*hGFAP*^ cKO mice. Moreover, blocking TBK1 signaling restored cellularity of the SVZ and DG areas of *Fip200*^*hGFAP*^ cKO mice (Fig. 7A–C), suggesting rescue of defective NSCs in these mice. Indeed, the pool of GFAP + Sox2 + NSCs as well as neurogenesis as marked by DCX and Ki67 staining were also rescued in the SVZ of *Fip200*^*hGFAP*^ cKO mice after TBK1 inhibition (Fig. 7D–F). These results are consistent with the hypothesis that aberrant activation of TBK1 and its subsequent phosphorylation of p62 is responsible for the increased p62 aggregates formation and NSC defects after the loss of non-canonical autophagy functions of FIP200. Moreover, amlexanox treatment reversed the increased ROS levels in the SVZ of *Fip200*^*hGFAP*^ cKO mice (Fig. 7G). Together, these results suggest that p62 aggregates formation triggered by TBK1 activation could lead to increased ROS that may also contribute to NSC defects in *Fip200*^*hGFAP*^ cKO mice.

## Discussion

In this study, we have taken a rigorous genetic approach to dissect the role and mechanisms of non-canonical functions of FIP200 in the regulation of NSC maintenance and differentiation by generation and analysis of a mouse model that specifically blocks its autophagy function (i.e. FIP200 cKI). Our results demonstrated that blocking induction of autophagy, similar to blocking autophagosome elongation by deleting Atg5 or Atg16L1^17^, did not lead to NSC defects, formally establishing that the loss of non-canonical functions of FIP200 was responsible for the defective NSC maintenance and differentiation. Moreover, our work reinforced the role of p62 aggregates, and identified TBK1 activation as the link for it, in causing NSC defects upon FIP200 deletion.

Our observations of TBK1 activation upon the loss of non-canonical functions of FIP200 in NSCs is consistent with a number of previous studies in other cells^28,37^. Goodwin et al. showed that FIP200 ablation increased TBK1 activation, which is required for ferritin degradation in an autophagy-independent manner in a number of cell lines^37^. In breast cancer cells, FIP200 deletion, but not specific blockade of FIP200 autophagy function, led to activation of TBK1^28^. However, our findings of a role for TBK1 activation as a major mediator for triggering p62-ubiquitinated protein aggregate formation and defective phenotypes in NSCs is distinct from these previous studies. For ferritin degradation, the activated TBK1 associated with another autophagy receptor TAX1BP1, rather than p62, and did not affect p62 levels or aggregation in several cell lines^37^. In breast cancer, activated TBK1 increased the expression of pro-inflammatory chemokines and infiltration of cytotoxic T cells^28^. While we also found increased p62 in breast cancer cells upon FIP200 deletion, this increase promoted tumor cell growth rather than being responsible for the reduced growth of FIP200-null tumor cells^38^. TBK1 was also identified as a contributing factor for selective autophagy by NBR1 that requires FIP200 but not autophagy genes for LC3 lipidation such as Atg5 and Atg7 in leukemia cell lines^39^. Although these studies including ours suggested TBK1 activation as a common theme upon FIP200 deletion, the consequences are varied in different cellular and biological settings. Nevertheless, it would be interesting to determine whether other TBK1 downstream targets, particularly other autophagy receptors such as TAX1BP1 and NBR1 in future studies.

Besides its core function in ULK1/Atg13/FIP200/Atg101 complex for autophagy induction through interaction with Atg13^27,40–43^, FIP200 has been shown to interact with other proteins to regulate diverse cellular functions^18^. Interestingly, a very recent structural and biochemical study identified a direct interaction of FIP200 with p62 through its C-terminal region (CT), which promotes degradation of p62-ubiquitinated protein aggregates^44^. These results raise the intriguing possibility that non-canonical functions of FIP200 can regulate NSC maintenance and neurogenesis through its direct interaction with p62 via the “Claw” domain, and loss of this interaction in FIP200-null NSCs could be responsible for p62 aggregate formation and NSC defects. Such a hypothesis is consistent with the suggestion that FIP200 plays dual roles in canonical autophagy and also serve as a scaffolding protein for selective autophagy of p62-ubiquitin aggregates or that by other autophagy receptors like NBR1^39^. Careful analysis of the dynamics of various autophagy machinery components and receptors in mitophagy (a proto-type of selective autophagy) also revealed a role for FIP200 coordinating with optineurin but upstream of Atg13 and potentially independent of its binding to Atg13^45^. This model suggesting a direct role for FIP200 interaction with p62 is not mutually exclusive with a function of TBK1 in mediating the process discussed above. In both studies on ferritin degradation and mitophagy^37,45^, TBK1 was shown to play an integral role in facilitating FIP200 interactions with autophagy receptors. It is interesting to note that TBK1 knockdown or its inhibitor reduced the size of p62 aggregates but not their numbers in our studies, suggesting that disruption of FIP200 interaction with p62 may be primary cause for its aggregate formation and activation of TBK1 amplifies the initial aggregates to trigger defects in NSCs. Moreover, increased p62 level upon FIP200 deletion, but not disruption of its interaction with Atg13 (see Fig. 2G), might also contribute to the p62 aggregation phenotype in cKO mouse brain. Lastly, it is also possible that the combination of the loss of non-canonical functions and its binding to Atg13 in FIP200-null NSCs are responsible for their reduced maintenance and differentiation. Future studies will be necessary to clarify these possibilities to gain mechanistic understanding for FIP200’s dual roles in canonical autophagy and non-canonical functions in NSCs and other appropriate physiological setting in vivo.

We noted that p62S403A mutant formed larger puncta in FIP200 KO cells than in WT cells. These results suggested that while S403A mutations significantly inhibited p62 puncta formation induced by TBK1 phosphorylation of this site in FIP200 KO cells, it did not completely eliminate puncta formation. It is possible that TBK1 could phosphorylate other minor sites in S403A mutant to induce its puncta formation at a low level. Alternatively, endogenous levels of p62 that can still be phosphorylated at this site may influence the puncta size of cells ectopically expressing p62(S403A). It will be interesting to determine these possibilities in future studies.

Although several studies including the current manuscript demonstrated TBK1 activation upon FIP200 deletion in different cells^46^, the mechanisms involved are not well understood at present. Our previous studies suggested that FIP200 interaction with a TBK1 adaptor molecule, AZI2, may serve to restrict TBK1 activation in other cells^28^. This FIP200-AZI2 interaction has been implicated in selective autophagy^47^ and thus the defective clearance of selective autophagy receptors such as p62 could lead to accumulation of TBK1 at these aggregates (positive feedback loop between p62 and TBK1). Since TBK1 activation can be achieved by trans-auto-phosphorylation, the accumulation of p62-TBK1 aggregates would ultimately result in unresolved TBK1 hyperactivation^48^. Thus, although the canonical-autophagy function of FIP200 is not involved in regulating TBK1, FIP200 may play additional non-canonical functions in selective autophagy through its C-terminus that is important for TBK1 regulation^39,44,47,49^. Future studies will be necessary to determine whether any of these mechanisms are involved in the increased phosphorylation of TBK1 upon FIP200 cKO in NSCs.

Oxidative damage is a major contributing factor for neurodegenerative diseases^50^, and increased ROS is associated with defective NSCs in *Fip200*^*hGFAP*^ cKO mice^16,17^. Previous studies showed that p62 can regulate a key antioxidative transcription factor Nrf2 though its binding to Keap1, leading to Nrf2 dissociating from Keap1 and translocating to the nucleus to activate its down-stream targets to orchestrate anti-oxidative responses^26,51^. Surprisingly, unlike the previous observations in liver cells^26^, we did not find increased Nrf2 activation despite increased p62 upon FIP200 deletion in NSCs^16,17^. Nevertheless, we could not exclude the possibility that inhibiting TBK1 could impact ROS levels independent of p62 aggregations on Nrf2 antioxidative activities. Future studies will also be necessary to understand the discrepancy in Keap1-Nrf2 response to p62 aggregates in NSCs versus liver cells.

Our previous studies showed that nestin-Cre induced *Fip200* deletion yielded not only defects similar to *Atg5* and *Atg7* deletions such as loss of Purkinje cells but also distinctive phenotypes including axonal swelling and spongiform degeneration not found in *Atg5* and *Atg7* conditional KO mice^13–15^. The progressive development of spongiosis in the white matters correlated temporally with the accumulation of p62-ubiquitinated aggregates and both were also observed in other regions of the brain. Although the underlying mechanisms are not fully understood, spongiform degeneration were due to extensive vacuolization in neuronal cells and associated with increased ROS and neurotoxicity in various degenerative disease models^52,53^. Thus, non-canonical functions of FIP200 could play important roles in neurons of other brain regions besides NSCs, and loss of such roles either alone or in combination with autophagy deficiency could have broader impact in various neurodegenerative diseases. In addition to the more widely appreciated autophagy modulation as potential therapies, further investigation of non-canonical functions of FIP200 and possibly other autophagy genes could lead to better understanding of mechanisms and potential treatment strategies for various neurodegenerative diseases.

## Methods

### Mice experiments

*Fip200*^*hGFAP*^ cKO mice with Fip200 conditionally deleted in postnatal NSCs (Wang et al.^16^) were generated by crossing hGFAP-Cre transgenic mice, expressing Cre in postnatal NSCs (Zhu et al., 2005; Wang et al.^16^), with FIP200 F/F mice. Alternatively, *Fip200*^*hGFAP*^ cKI mice carried the FIP200 F/KI alleles (Chen et al.^27^) and were crossed with hGFAP-Cre mice. All mice were maintained on C57BL/6 backgrounds. Age- and littermate-matched control and mutant mice were used for analysis to minimize the impact of modifier genes. Mice were housed and handled according to local, state, and federal regulations. All experimental procedures were performed according to the guidelines of the Institutional Animal Care and Use Committee of the the University of Cincinnati (protocol #19-09-18-02, approval date 12/3/19). Genotyping for Fip200f./f and FIP200 + /KI and Cre alleles was performed by PCR analysis of tail DNA, essentially as described previously (Wang et al.^16^, Chen et al.^27^).

### Antibodies and reagents

Primary antibodies used were: mouse anti-GFAP (Thermo Fisher Scientific), mouse anti-nestin (Rat-401), mouse anti-p62 (Abcam), rat anti-phosphorylated p62 (MBL: D343-3), mouse anti-ubiquitin (Santa Cruz Biotechnology), rabbit anti-TBK1 (Cell Signaling), rabbit anti- phosphorylated TBK1 (Novus Biologicals), rabbit anti-GFAP (Dako), rabbit anti-Ki67 (Spring Bioscience), rabbit anti-p62 (Enzo), rabbit anti-Sox2 (Millipore), rat anti-Ki67 (BioLegend), and guinea pig anti-doublecortin (anti-DCX; EMD Millipore). Secondary antibodies were goat anti–rabbit IgG-FITC, goat anti–rabbit IgG–Texas red, goat anti–mouse IgG-FITC, goat anti–mouse IgG–Texas red, goat anti–mouse IgG-HRP, and goat anti–rabbit IgG-HRP (Jackson Immunology). Dihydroethidium (DHE) was purchased from Sigma-Aldrich and Amlexanox was purchased from MedChemExpress. Transfections were carried out using Lipofectamine 3000 reagent (Invitrogen).

### Plasmid constructs and siRNA

HA-TBK1 plasmid was kindly provided by Dr. Shitao Li. Plasmid p62-mCherry was purchased from addgene (# 55,132) Mutation of TBK1(K38M) and p62(S403A) were constructed using NEB mutagenesis kit according to manufacturer’s instructions (NEB# E0552S). TBK1 siRNAs were purchased from Dharmacon (J-003788-08-0005, J-003788-09-0005). Plasmids and siRNAs were introduced into cells with Lipofectamine 3000 transfection reagent.

### Protein extraction, SDS-PAGE, and immunoblotting

Protein extraction from neurospheres and cell cultures was carried out as described previously^16,17^. Cells were lysed by RIPA buffer (50 mM Tris–HCl, pH 7.4, 1% Triton X-100, 0.2% sodium deoxycholate, 0.2% SDS, and 1 mM sodium EDTA) and supplemented with Halt Protease Inhibitor Cocktail (Thermo Scientific). Protein lysates were collected after centrifugation and concentrations were determined using Bio-Rad protein assay reagent. Lysates were boiled for 5 min in 2 × SDS sample buffer (50 mM Tris–HCl, pH 6.8, 12.5% glycerol, 1% SDS, and 0.01% bromphenol blue) containing 5% β-mercaptoethanol and analyzed by SDS-PAGE followed by immunoblotting using various antibodies.

### Neurosphere and cell culture

Neurospheres were cultured as described before (Wang et al.^16^). In brief, the SVZ cells of mice at P0 were isolated under a dissection microscope and cut into ∼1-mm3 cubes. 0.2% trypsin was used to digest the tissues to obtain single-cell suspensions. The cells were cultured in neurobasal medium supplemented with B27 (Invitrogen), 10 ng/ml bFGF, and 20 ng/ml EGF (Invitrogen) in Ultra-Low Attachment dishes (Corning). Medium was supplemented on day 3, day 6 and day 9. After 9–10 days of culturing, neurospheres were collected for subsequent experiments. HeLa cells were obtained from ATCC and maintained in DMEM supplemented with 10% FBS. HeLa FIP200 KO cells were generated as described previously (Hao et al., 2021).

### Histology and immunofluorescence (IF)

Histology and immunofluorescence of brain sections were performed using protocols as described previously^16,17^. For tissue collection, mice were euthanized with CO2 and hole brain was collected after perfusion. After fixation for 16 h at 4 °C using 4% (wt/vol) freshly made, prechilled PBS-buffered PFA, brain tissues were sagittal sectioned, embedded in paraffin, and sectioned at 5 µm with a Leica 2125 Microtome (Leica). For H&E staining, sections were stained with hematoxylin and eosin (H&E) for routine histological examination. Comparable positions (triangular lateral ventricle with intact RMS) were examined under a BX41 light microscope (Olympus) equipped with a 20 × 0.5 NA UPlanFl air objective lens, and images were captured at room temperature with a digital camera (model DP70; Olympus) using DP Controller software (Version 1.2.1.10 8). For brain sections, IF was performed firstly in three washes of xylene (3 min each) and then rehydrated in graded ethanol solutions (100%, 95%, 70%, 50%, and 30%, 1 min each). After heat-activated antigen retrieval (Retriever 2000; PickCell Laboratories), Protein Block Serum Free (Dako) was used to treat the sections at room temperature for 10 min. Then primary antibodies were incubated at 4 °C for 16 h. After incubation, sections were washed in PBS three times (5 min each) and incubated with 1:200 FITC or Texas red–conjugated secondary antibodies for 1 h at room temperature. Three times wash (5 min each) was performed in PBS after incubation. DAPI was stained for nuclei and Vectashield mounting medium (Vector Laboratories) was used the mount the sections.

For IF of cultured cells, prechilled 4% PFA in PBS was firstly used to fix cells for 10 min. Then cells were permeabilized with 0.1% Triton in PBS before blocking with 3% BSA (Thermo Fisher) in PBS. Primary and secondary antibodies were incubated as described as above.

### Amlexanox and DHE administration

Amlexanox (25 mg/kg) was injected i.p. daily from day 16 to day 28. Brain tissues were embedded in paraffin and sectioned at 5 µm for further staining. DHE (27 mg/kg) was injected intraperitoneally 18 h before the mice were perfused with 4% PFA in PBS (Wang et al.^16^). Brain samples were postfixed in 4% PFA for 1 week and treated with 20%, 30% sucrose overnight at 4 °C. 10 µm cryosection was performed in 3050 Cyrostat machine (Leica, Germany). For DHE stained samples, IF was carried out as described above.

### Laser scanning confocal microscope imaging and Statistical analysis

Confocal image was acquired at room temperature using an Axiovert 710 Meta laser scanning confocal microscope (Carl Zeiss) equipped with a 63 × /1.2 water-corrected oil-immersion lens. Images were integrated and processed using Zen 2010 software. All data were processed identically between samples. Lengths, areas, and the number of cells from comparable sections were quantified using ImageJ software. For quantification in SVZ area, three 200 × 200(w × h) areas were chosen by Oval selections in ImageJ for each mice. Statistical significance was evaluated by Student’s t test, with *P* < 0.05 indicative of statistical significance using Graph Pad Prism (version 7.0). The number of animals used for quantification is indicated in the figure legends. Data were plotted as means ± SEM.

### Statement

All the studies in the manuscript were designed and performed in accordance with ARRIVE guidelines 2.0.

## Supplementary Information

Supplementary Information.
