# Supplementary material for: Non-canonical function of FIP200 is required for neural stem cell maintenance and differentiation by limiting TBK1 activation and p62 aggregate formation
Source: Sci Rep. 2021 Dec 13;11:23907. doi: 10.1038/s41598-021-03404-7 (PMC8668875; doi:10.1038/s41598-021-03404-7)

**Supplementary Information**

**Fig. S1: Full length blots corresponding to figure 2G.**

Full length immunoblots showing levels of FIP200, TBK1, pTBK1, p62, pp62 (S403) and GAPDH in lysates extracted from neurospheres of Ctrl, *Fip200^hGFAP^* cKO and and *Fip200^hGFAP^* cKI mice.


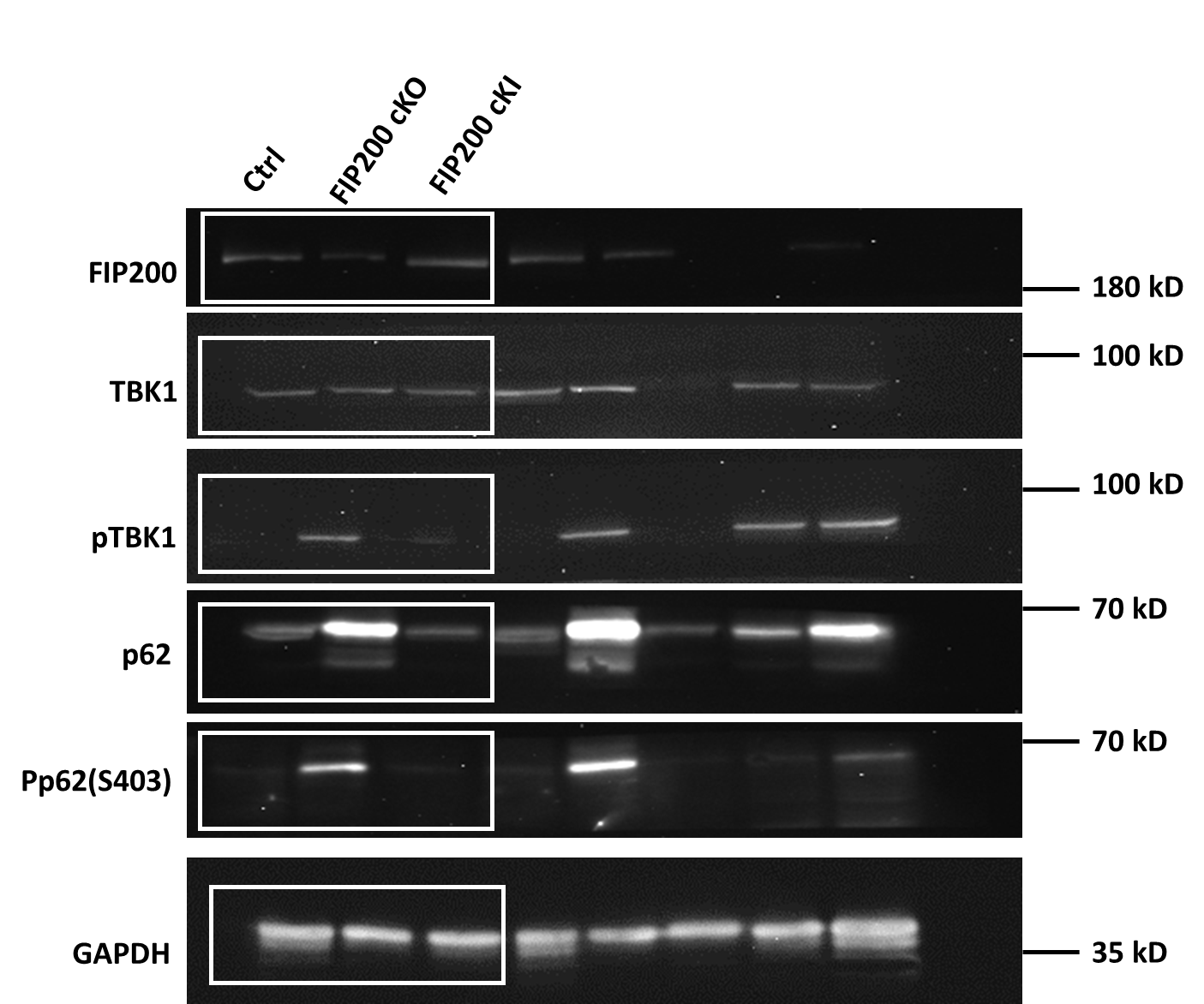


**Fig. S2: Full length blots corresponding to figure 5C.**

Full length immunoblots showing levels of FIP200, TBK1, p62, pp62 (S403) and GAPDH in WT and FIP200 KO HeLa cells with or without TBK1 siRNA transfection.


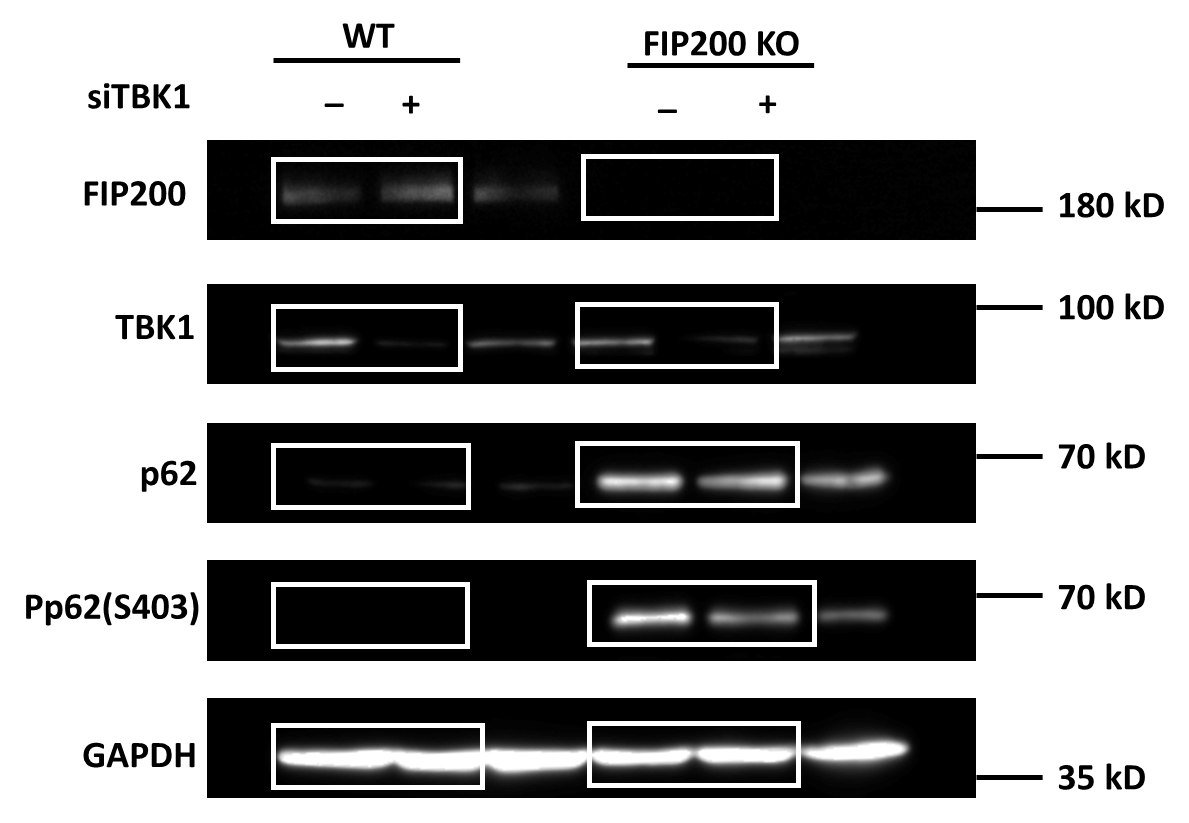

Supplement: Supplementary file 1 — Supplementary Information. [file 41598_2021_3404_MOESM1_ESM.docx]
